# Analysis of the Antimicrobial and Anti-Biofilm Activity of Natural Compounds and Their Analogues against *Staphylococcus aureus* Isolates

**DOI:** 10.3390/molecules27206874

**Published:** 2022-10-13

**Authors:** Sobia Mastoor, Fizza Nazim, Syed Rizwan-ul-Hasan, Khalid Ahmed, Shabnam Khan, Syed Nawazish Ali, Syed Hani Abidi

**Affiliations:** 1 Department of Chemistry, Faculty of Science, University of Karachi, Karachi 75270, Pakistan; 2 Department of Pharmaceutical Chemistry, Faculty of Pharmacy, Hamdard University, Karachi 74600, Pakistan; 3 Department of Biological and Biomedical Sciences, Aga Khan University, Karachi 74800, Pakistan; 4 Department of Computer Science, DHA Suffa University, Karachi 75500, Pakistan; 5 Department of Microbiology, University of Karachi, Karachi 75270, Pakistan; 6 Department of Biomedical Sciences, Nazarbayev University School of Medicine, Nur-Sultan 010000, Kazakhstan

**Keywords:** *Staphylococcus aureus*, natural compounds, anti-biofilm, antimicrobial, gene expression

## Abstract

(1) Background: *Staphylococcus aureus (S. aureus)* is one of the most frequent causes of biofilm-associated infections. With the emergence of antibiotic-resistant, especially methicillin-resistant *S. aureus* (MRSA), there is an urgent need to discover novel inhibitory compounds against this clinically important pathogen. In this study, we evaluated the antimicrobial and anti-biofilm activity of 11 compounds, including phenyl propenes and phenolic aldehydes, eugenol, ferulic acid, sinapic acid, salicylaldehyde, vanillin, cinnamoyl acid, and aldehydes, against drug-resistant *S. aureus* isolates. (2) Methods: Thirty-two clinical *S. aureus* isolates were obtained from Alkhidmat Diagnostic Center and Blood Bank, Karachi, Pakistan, and screened for biofilm-forming potential, and susceptibility/resistance against ciprofloxacin, chloramphenicol, ampicillin, amikacin, cephalothin, clindamycin, streptomycin, and gentamicin using the Kirby-Bauer disk diffusion method. Subsequently, 5 representative clinical isolates were selected and used to test the antimicrobial and anti-biofilm potential of 11 compounds using both qualitative and quantitative assays, followed by qPCR analysis to examine the differences in the expression levels of biofilm-forming genes (*ica*-A, *fnb*-B, *clf*-A and *cna*) in treated (with natural compounds and their derivatives) and untreated isolates. (3) Results: All isolates were found to be multi-drug resistant and dominant biofilm formers. The individual Minimum Inhibitory Concentration (MIC) of natural compounds and their analogues ranged from 0.75–160 mg/mL. Furthermore, the compounds, Salicylaldehyde (SALI), Vanillin (VAN), α-methyl-*trans*-cinnamaldehyde (A-MT), and *trans*-4-nitrocinnamic acid (T4N) exhibited significant (15–92%) biofilm inhibition/reduction percentage capacity at the concentration of 1–10 mg/mL. Gene expression analysis showed that salicylaldehyde, α-methyl-*trans*-cinnamaldehyde, and α-bromo-*trans*-cinnamaldehyde resulted in a significant (*p* < 0.05) downregulation of the expression of *ica*-A, *clf*-A, and *fnb*-A genes compared to the untreated resistant isolate. (4) Conclusions: The natural compounds and their analogues used in this study exhibited significant antimicrobial and anti-biofilm activity against *S. aureus*. Biofilms persist as the main concern in clinical settings. These compounds may serve as potential candidate drug molecules against biofilm forming *S. aureus*.

## 1. Introduction

*Staphylococcus aureus* (*S. aureus*) is a gram-positive, biofilm-forming pathogen most commonly associated with community- and hospital-acquired infections [1]. The biofilm-forming ability of this bacterium limits the efficacy of antimicrobial agents, which contributes to the severity of the infection and may worsen the outcomes of the disease (e.g., cystic fibrosis), thereby posing an immense clinical challenge [2].

*S. aureus* can strongly adhere to natural and abiotic surfaces as it encodes proteins that mediate adherence to host tissues and surfaces, and consequently forms mechanically and chemically robust biofilms [3]. *S. aureus* is mainly prominent for the abundance of microbial adhesion molecules, known as Microbial Surface Component Recognizing Adhesive Matrix Molecules (MSCRAMMs) [4]. These adhesion proteins comprise intracellular adhesion (*ica-A*), collagen-binding adhesion (*cna*), clumping factors A and B (*clf-A and clf-B*), fibronectin-binding proteins (*fnb*), etc. [5]. Even though the biofilm development in *S. aureus* is influenced by a variety of variables, polysaccharide intercellular adhesins (PIA), which are encoded by the *ica* operon, play the most significant role [6].

Biofilm formation also contributes to antibiotic resistance and tolerance to immune cells [7]. Methicillin-resistant *S. aureus* (MRSA), in particular, poses a greater risk, which causes septicemia in clinical settings [8]. The antibiotic-resistant isolates are hard to treat and a common cause of *S. aureus*-related mortalities. The emergence of antibiotic resistance warrants a search for alternate inhibitory agents. One of the effective alternate sources is medicinal plants that have been utilized for centuries for the treatment of infectious diseases [9]. Natural compounds from the plants, such as phenylpropanoid, eugenol, cinnamic acid, and cinnamaldehyde, and their similar scaffolds have traditionally been used for treating infections [10,11].

In the present study, we examined the antimicrobial and anti-biofilm potential of 11 natural compounds, including aromatic aldehyde, substituted cinnamaldehyde, α-methyl-cinnamic acid, hydroxy-cinnamic acid, *trans*-4-nitrocinnamic acid, and 4-allyl-2-methoxyphenol. Since *S. aureus* is known to express adhesion- and virulence-related genes, we also used qPCR to analyze the differences in the expression levels of biofilm-forming genes (*ica-A, fnb-B, clf-A,* and *cna)* with and without natural compound treatment.

## 2. Results

### 2.1. Antibiotic Resistance/Susceptibility Profile of S. aureus Isolates

The antibiotic resistance/susceptibility profile revealed most of the isolates to be multi-drug resistant, wherein the highest resistance, i.e., 53.1%, and 34.3%, was observed against ciprofloxacin and ampicillin, respectively (Table 1). On the contrary, 93.7% and 90.6% of the *S. aureus* isolates were found to be sensitive to gentamicin and chloramphenicol, respectively (Table 1).

### 2.2. Analysis of Biofilm-Forming Capacity of S. aureus Isolates

**Figure 1:**
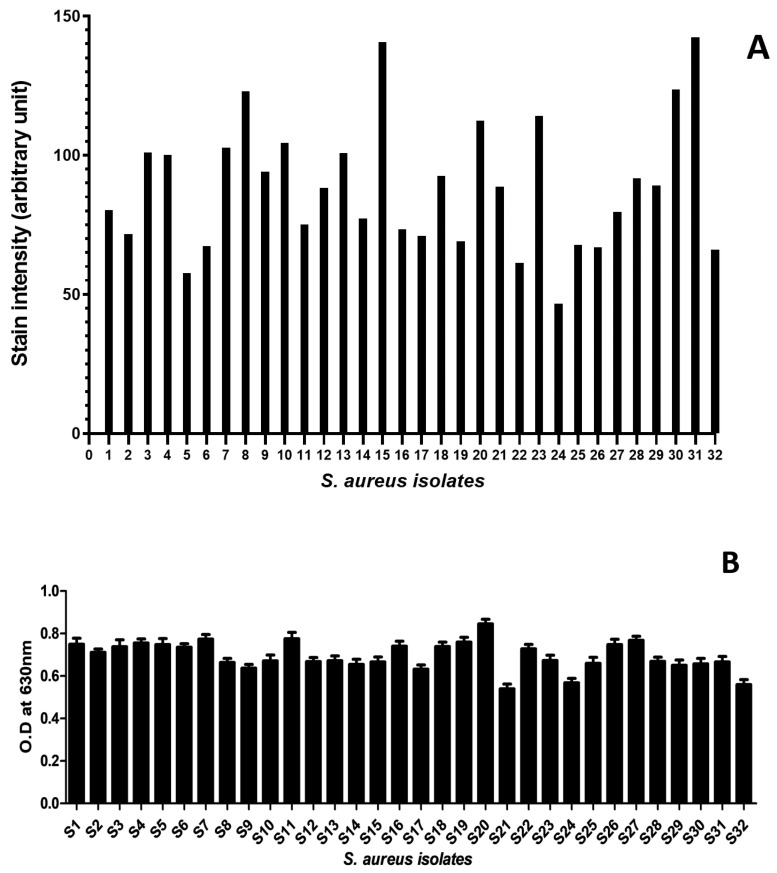
**Qualitative and quantitative analysis of biofilm formation by *S. aureus* isolates:** (**A**) biofilm staining intensity, for each isolate, measured using ImageJ software. The plot shows mean staining intensity (arbitrary unit) at the *Y*-axis, while the *X*-axis shows the isolates tested (S1–S32). (**B**) spectrophotometric analysis (OD630 nm) for the biofilm formation for each isolate. The plot shows mean OD values with standard deviation as an error bar on *Y*-axis, while isolates on *X*-axis.

The biofilm-forming capacity of *S. aureus* isolates was qualitatively analyzed using the air–liquid coverslip assay. The analysis revealed dense biofilm staining and visible microbial aggregation on the coverslips, suggesting the isolates to be potent biofilm formers (Supplementary Figure S1). The image analysis (quantitative analysis of crystal violet-stained biofilms) also showed the mean crystal violet stain intensity (proportional to the amount of biofilm formed [12,13]) to be 88.72 (57.67 to 142.33; Supplementary Table S1), indicating a heavy stain uptake and a high amount of biofilm formation by all isolates (Figure 1A). The observation was supported by the quantitative microtiter plate assay, which showed the OD_630nm_ for all isolates to be above 0.5 (0.5–0.9), suggesting a strong biofilm-forming potential for each isolate (Figure 1B). The Pearson correlation analysis showed a very weak positive correlation (r = 0.06) between the measured optical density and the biofilm stain color intensity (quantified from biofilm images).

### 2.3. Minimum Inhibitory Concentration (MIC) and Anti-Biofilm Activity of Test Compounds *(**I–XI**)* against S. aureus Isolates

Based on the resistance/susceptibility pattern, 5 isolates, 2 were susceptible (S11 & S12) to all antibiotics, 2 were resistant (S24 & S25) against all antibiotics, and 1 (S31) exhibited intermediate resistance to all antibiotics, were selected to be used in the assay to determine the MIC of the 11 test compounds (Table 2). Out of the 11 compounds, α-bromo-*trans*-cinnamaldehyde (A-BT) and α-methyl-cinnamic acid (A-MCA) exhibited the lowest MIC range (1–5 mg) against all isolates, followed by 4-acetoxy-3-methoxycinnamaldehyde (4A3M) and salicylaldehyde (SALI), which exhibited MICs in the range of 1–20 mg/mL and 1–30 mg/mL, respectively, in all isolates (Table 2). It is important to mention that all compounds exhibited bactericidal activity as no growth was observed on agar plates when culture media from the MIC wells was plated onto the agar plates.

Subsequently, we used a quantitative biofilm assay to determine the anti-biofilm activity of all 11 test compounds. Overall, all the compounds exhibited 15–100% inhibition/reduction in biofilm formation at the concentration of 1–10 mg/mL (Table 2). The highest anti-biofilm activity, ranging from 70–93%, was exhibited by salicylaldehyde (SALI), vanillin (VAN), α-methyl- *trans*-cinnamaldehyde (A-MT), and *trans*-4-nitrocinnamic acid (T4N).

### 2.4. Effect of Test Compounds on Expression of Biofilm-Associated Genes

**Figure 2:**
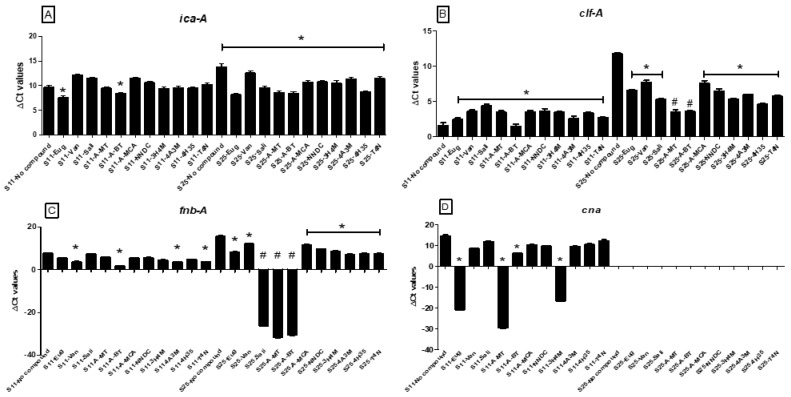
**Expression of genes associated with biofilm formation in treated and untreated samples.** The expression (∆Ct) of (**A**) *ica*-A, (**B**) *clf*-A, (**C**) *fnb*-A, and (**D**) *cna* genes in sensitive (S11) and resistant (S25) isolates with and without all 11 test compounds treatment. The asterisk * indicates *p*-value < 0.05 and # indicates *p*-value <0.001.

In the next step, we examined the effect of the 11 natural compounds on the expression of *ica*-A, *clf*-A, *cna*, and *fnb*-A genes (associated with biofilm formation) [14] in resistant and sensitive isolates. An analysis of baseline (untreated) expression showed the expression of all but *cna* genes in the resistant isolate, while in the sensitive isolate, the expression of all genes was observed (Figure 2).

For the resistant isolate, treatment with all eleven compounds, particularly salicylaldehyde, α-methyl-*trans*-cinnamaldehyde, and α-bromo-*trans*-cinnamaldehyde, resulted in a significant decrease in the expression of *ica-A* (SALI = 9.95, A-MT = 8.25, A-BT = 8.75; *p* < 0.05), *clf*-A (SALI = 5.4; *p* < 0.05, A-MT = 3.09, A-BT = 3.65; *p* < 0.01) and *fnb*-A genes (SALI = −26.45, A-MT = −31.51, A-BT = 30.00 respectively; *p* < 0.01) as compared to the untreated resistant isolate (*ica-A*: 14.35, *clf*-A: 11.51 and *fnb*-A: 15.44) (Figure 2).

For the sensitive isolate, out of 11 natural compounds, only the treatment with eugenol (7.47; *p* < 0.05) and α-bromo-*trans*-cinnamaldehyde (8.6; *p* < 0.05) demonstrated a significant reduction in the expression of *ica-A* gene (Figure 2). Similarly, the expression of the *cna* gene was significantly reduced when treated with eugenol (−20.54; *p* < 0.001), α-methyl- *trans*-cinnamaldehyde(−29.09; *p* < 0.001), α-bromo-*trans*-cinnamaldehyde (6.60; *p* < 0.05), and 3-hydroxy-4-methoxycinnamic acid (−16.73; *p* < 0.001), while the expression of only the *clf-A* gene was significantly increased (1.79–4.27; *p* < 0.05) in the treated samples as compared to the untreated sample (*clf-A* = 1.08) (Figure 2).

## 3. Discussion

In this study, the antibiotic resistance/susceptibility profiling showed that most of the clinical isolates were resistant to ampicillin (34.3%) and ciprofloxacin (53.1%). This observation is supported by earlier studies showing a high prevalence of ampicillin- and ciprofloxacin-resistant strains of *S. aureus* and MRSA [15,16,17,18,19].

In the next step, biofilm formation was tested in all isolates (S1–S32) using the air–liquid interface coverslip assay (qualitative) and microtiter plate assay (quantitative). Studies have used static biofilm systems, such as air–liquid interphase assays, in combination with the microtiter plate assay since it allows direct visualization of the biofilm-forming potential of the studied pathogens [20]. The air–liquid interphase assay provides an opportunity to observe biofilm formation on surfaces, such as glass [21], whereas the microtiter plate spectrophotometric assay provides an indirect way to quantitatively assess the biofilm formation potential [22]. In our study, the images from the air–liquid coverslip assay showed dense staining of the microbial aggregation, which is consistent with biofilm formation (Supplementary Figure S1), while the image analysis of the stained coverslips offered quantification of biofilms based on color intensity and revealed strong biofilm by all clinical isolates (Figure 1A). It is important to mention that air–liquid interphase assay has a few limitations, the main one being that it provides a relatively crude estimate of biofilm formation, when in contrast, sophisticated techniques are available to analyze biofilms, including those which involve the analysis of specific biofilm proteins [23,24,25]. However, since our laboratory did not have those capabilities, we used the combination of air–liquid interphase assay and microtiter plate assay. Furthermore, to ameliorate some of the limitations, and to better characterize the results from the air–liquid interphase assay, we quantified—using ImageJ software—the staining intensity from each image, where the staining intensity was proportional to the amount of biofilm formed [12,13]. The findings were corroborated by the quantitative microtiter plate assay showing OD_630nm_ of all isolates to be greater than 0.5 (Figure 1B). Studies have reported that isolates exhibiting OD > 0.5 are considered strong biofilm formers [26,27,28]. The microtiter plate assay is a standard assay used by numerous studies to characterize biofilm-forming pathogens as strong or weak biofilm producers [26,27,28].

In the next step, we tested the antimicrobial and anti-biofilm activity of 11 compounds, including 2 phenolic aldehydes, 4 substituted cinnamaldehydes, 2 hydroxy-cinnamic acids, α-methyl-cinnamic acid, trans-4-nitrocinnamic acid, and 4-allyl-2-methoxyphenol. These phytochemicals, many of which are secondary metabolites—for example, phenyl propanoids (cinnamic acid, ferulic acid, sinapic acid and eugenol) and phenolic compounds (salicyclaldehyde and vanillin)—have been reported in several species of plants as bioactive molecules [29,30,31,32,33]. For instance, eugenol is a major phenolic constituent of the clove plant *Syzygium aromaticum*, family Myrtaceae [34], and it is a phenyl propanoid (C6-C3) derived from guaiacol and naturally occurs in dietary plants as well as medicinal herbs, and plays a significant role in preventing drug resistance [35]. Cinnamaldehyde is a major phytochemical of cinnamon essential oil, and it occurs naturally in the bark and leaves of the *Cinnamoum zeylanicum blume*, which is a medicinal plant from family Lauraceae. Cinnamaldehyde is found to be efficacious against biofilm formation [36]. Salicylaldehyde (2-hydroxybenzaldehyde) can be extracted from groats of buckwheat, *Fagopyrum esculentum* family Polygonaceae. Natural vanillin is a composite blend of different phytoconstituents, and is found in the plant kingdom, genus Vanillus, in the pods of different plant species, i.e., *V. planifolia, V. tahitensis*, and *V. pompon* [37]. Sinapic acid (4-hydroxy-3, 5-dimethoxycinnamic acid) is a natural product extensively present in vegetables (kale, white cabbage, turnip, and broccoli), spices (anise, rosemary, sage, and borage), berries (strawberry, cranberry, and blueberry), oilseed crops (rapeseed), and lemon [38,39]. Sinapic acid has also been reported as an anti-inflammatory and antibacterial agent with anti-biofilm activity [40]. Naturally-occurring hydroxy cinnamic acid belongs to the class phenolic compounds (sinapic acid, ferulic acid, and caffeic acid), and these bioactive molecules have been reported as phenolic antioxidants [41].

The results of this study showed that α-bromo-trans-cinnamaldehyde (A-BT) and α-methyl-cinnamic acid (A-MCA) exhibited the lowest MIC range (1–5 mg/mL) against the tested isolates. This finding is supported by earlier studies where cinnamaldehyde and its derivatives have been shown to have anti-biofilm and antimicrobial activity against *S. aureus* with MIC against growth at 100 µg/mL [42]. Similarly, in this study, the highest anti-biofilm activity, ranging from 70–93%, was exhibited by salicylaldehyde (SALI), vanillin (VAN), α-methyl-*trans*-cinnamaldehyde (A-MT), and *trans*-4-nitrocinnamic acid (T4 N). Studies have reported that Schiff-based compounds are derived from salicylaldehyde contain potent antimicrobial activity [43,44], however, our study reveals the anti-biofilm potential of salicylaldehyde against *S. aureus*. In addition, previous studies have provided evidence that essential oils containing vanilla exhibited strong inhibitory activity against *S. aureus* biofilms on catheters [45]. Similarly, studies have reported the bactericidal and anti-biofilm effects of cinnamaldehyde against *S aureus* [46]. It has also been reported that nitrocinnamic acid inhibits the growth of sessile and planktonic forms of *S. aureus* [47], however, our study provides evidence for the anti-biofilm potential of nitrocinnamic acid against *S aureus.*

A gene expression analysis of biofilm-associated genes (*ica*-A, *clf*-A, *cna*, and *fnb*-A) showed that in resistant isolates, the treatment with salicylaldehyde, α-methyl-*trans*-cinnamaldehyde, and α-bromo-*trans*-cinnamaldehyde resulted in significant decrease in the expression of *ica-A, clf*-A, and *fnb*-A genes in natural compound-treated isolates. Whereas, in the sensitive isolates, only treatment with eugenol and α-bromo-*trans*-cinnamaldehyde demonstrated a significant reduction in the expression of the *ica-A* gene. Similarly, the expression of the *cna* gene was significantly reduced when treated with eugenol, α-methyl-*trans*-cinnamaldehyde, α-bromo-*trans*-cinnamaldehyde, and 3- hydroxy-4-methoxycinnamic acid. Since *ica*-A and its protein-polysaccharide intercellular adhesin play an important role in biofilm formation in *S. aureus* [48], the decrease in its gene expression in the treated group might explain the mechanism of action of these compounds. In addition, *clf-A* and *fnb*-A, like other MSCRAMMs, are adhesin proteins that mediate the initial attachment of bacteria with the surfaces and are expressed in all biofilm-forming *S. aureus* isolates [49,50]; therefore, a decrease in its expression, as shown in this report, provides insights into the targeted mechanism of the compound in inhibition of biofilm formation. The gene expression of *fnb-A* has also been found to be associated with *S. aureus* isolates, which are strong biofilm formers [51], thereby indicating it to be a major virulence factor in this pathogen. Similarly, *S. aureus-*associated arthritis involves the adherence and colonization of the joint cartilages by the pathogen, and it has been shown that it requires the enhanced expression of the *Cna* gene (adhesion gene) in *S. aureus* [52]. Although our study provides some insights into the mechanism of action of the natural compounds used in the study, further functional studies are required to fully understand the mechanism through which these natural compounds exhibit antimicrobial and anti-biofilm activity.

In summary, biofilms persist as the main concern in clinical and industrial fields. This study indicates that natural compounds and their analogues can act as potential antimicrobial agents with the ability to block adhesion and biofilm formation in *S. aureus*.

## 4. Materials and Methods

### 4.1. S. aureus Isolates

For this study, we obtained 32 laboratory-confirmed clinical *S. aureus* isolates from Alkhidmat Diagnostic Center and Blood Bank, Karachi, Pakistan. The isolates were labeled as S1–S32. The isolates were cultured and maintained in Tryptic Soy Broth (TSB; Oxoid) at 37 °C.

### 4.2. Kirby-Bauer Disc Diffusion Assay for Determination of Antibiotic Susceptibility/Resistance Pattern for S. aureus Isolates

As per the guidelines of the Clinical and Laboratory Standards Institute 2015 [30], a Kirby-Bauer disc assay was used for the analysis of antibiotic susceptibility/resistance pattern for *S. aureus* isolates against 8 clinically prescribed antibiotics, namely Gentamicin (CN) 120µg, Chloramphenicol (C) 30 µg, Cephalothin (KF) 30 µg, Amikacin (AK) 30 µg, Ampicillin (AMP) 10 µg, Streptomycin (S) 10 µg, Ciprofloxacin (CIP) 5 µg, and Clindamycin (DA) 2 µg. For this assay, a lawn of *S. aureus* was prepared with 0.5 McFarland (10^8^ CFU/mL) cultures on nutrient agar plates. Commercial disks (Oxoid^TM^, Thermo Fisher Scientific, Horsham, UK) of the above-mentioned antibiotics were placed on the agar with the help of sterile forceps, and the plates were incubated at 37 °C for 24 h. Following incubation, the zones of inhibition were measured in millimeters.

### 4.3. Preparation of Stock Solutions of Natural Compounds

For this study, we used the following compounds (**I**–**XI**): 2-hydroxybenzaldehyde/salicylaldehyde (SALI), 3-methoxy-4-hydroxybenzaldehyde/Vanillin (VAN), α-methyl- *trans*-cinnamaldehyde (A-MT), α-bromo-*trans*-cinnamaldehyde (A-BT), *N*, *N*-dimethyl-cinnamaldehyde (NNDC), 4-acetoxy-3-methoxycinnamaldehyde (4A3M), α-methyl-cinnamic acid (A-MCA), 3-hydroxy-4-methoxycinnamic acid (3H4M), 4-hydroxy-3,5-dimethoxycinnamic acid (4H35), and *trans*-4-nitrocinnamic acid (T4N). All chemicals were purchased from Alfa Aesar (Thermo Fisher Scientific, Waltham, MA, USA), except 4-allyl-3-methoxyphenol/Eugenol (EUG), which was obtained from Daejung Chemicals (Siheung-si, South Korea). The stock solutions of all compounds were prepared in pure DMSO (>99.7%).

### 4.4. Determination of Biofilm Forming Potential of the S. aureus Isolates

The biofilm-forming potential of *S. aureus* isolate was evaluated using the air–liquid interface coverslip assay and the microtiter plate spectrophotometric assay [31,32].

#### 4.4.1. Air–Liquid Interface Assay

For the air–liquid interface assay, 20 µL of *S. aureus* cultures, matching the 0.5 McFarland index, were inoculated in 3 mL of TSB in each well of sterile 12-well plates. A coverslip was cautiously placed in each well at 90° angle to the base of the well and incubated at 37° C for 48 h. After incubation, coverslips were washed with distilled water and placed in a new 12-well plate containing 4 mL of crystal violet (0.1% *w*/*v*) solution in each well. The coverslips were stained for 15 min. Subsequently, the coverslips were washed with distilled water three times and dried on a hotplate. The coverslips were visualized under a high-power light microscope (Olympus BX43, Tokyo, Japan), and images were captured using a digital camera [33]. We also quantified the staining intensity using ImageJ 1.52 software. For this purpose, in each image, a representative region of interest (ROI = stained biofilm areas) was selected using the polygon selection tool in the ImageJ software. The areas in the images where biofilm formation was absent or there was a staining artifact were not included in the analysis. Using color histograms of the RBG images plugin, the mean values of color intensity, namely red (rMean), green (gMean), and blue (bMean), were obtained and the average scores of these means were calculated with standard deviations, resulting in a number indicating the overall stain intensity in the regions of interest in each image.

#### 4.4.2. Spectrophotometric Assay for Quantitative Analysis of Biofilm Formation

For this assay, 2 µL of *S. aureus* cultures, matching the 0.5 McFarland index, were added into 200 µL (Tryptone Soy Broth) TSB in each well of a 96-well untreated flat-bottom microtiter plate (Thermo Scientific™ Nunc™, Waltham, MA, USA) and incubated at 37 °C for 24 h. One well, containing TSB only, served as a negative control or blank. Subsequently, the absorbance (optical density [OD]) was measured at 630 nm using a Multiskan Sky Microplate Spectrophotometer (Agilent Technologies, Palo Alto, CA, USA). OD-based scoring for biofilm formation was done as follows: Non/weak = 0–0.5, moderate = 0.5, and strong biofilm = >0.5 [32,34].

#### 4.4.3. Analysis of Correlation

A Pearson correlation analysis was conducted between the measured mean values of the optical density and the biofilm stain color intensity to analyze the relationship of optical density with the biofilm color intensity using GraphPad 8.4.3 software.

### 4.5. Determination of Minimum Inhibitory Concentration of Compounds *(**I–XI**)*

The minimum inhibitory concentrations (MIC) of the natural compounds were determined using untreated, sterile 96-well plates containing 150 µL TSB and 2 µL fresh overnight culture of *S. aureus*. Blank wells contained only media, while the control wells contained only *S. aureus* culture and TSB. The test wells contained *S. aureus* culture and all test compounds at different concentrations, ranging from 0.1–500 mg. The plate was incubated at 37 °C for 24 h in a stationary condition. Subsequently, the MIC value for each of the 11 compounds was determined, wherein the well exhibiting no visible bacterial growth was considered as MIC well. The experiments were performed in duplicate.

#### Determination of Bactericidal/Bacteriostatic Effect of Compounds (**I–XI**)

For confirmation of the MIC and determination of bactericidal/bacteriostatic effect of compounds, the culture media from the MIC wells (Table 2) was streaked onto the nutrient agar plate and observed for growth.

### 4.6. Determination of the Inhibitory Activity of the Test Compounds *(**I–XI**)* against S. aureus Biofilms

A quantitative evaluation of the anti-biofilm activity of 11 compounds was performed using a microtiter plate spectroscopic assay [33,34]. It is one of the most widely used methods to test the antimicrobial and anti-biofilm activity of compounds [53,54]. Briefly, fresh cultures of *S. aureus* isolates were prepared in 5 mL of TSB and incubated overnight at 37 °C. After incubation, *S. aureus* cultures were diluted (1:100) in TSB, and 100 µL of each diluted culture was added to each well of a 96-well untreated, flat-bottom microtiter plate (Thermo Scientific™ Nunc™, Waltham, USA). In the control well, solvent (DMSO) was added in the same concentration as used in the compound. In the test wells, compounds were added to MIC concentrations and incubated at 37 °C for 24 h. The next day, the contents of the wells were discarded by aspiration, and wells were gently washed with autoclaved distilled water. Subsequently, the wells were stained with 250 µL crystal violet (0.1% *w*/*v*) and incubated for 15 min at room temperature with the lid closed. Following incubation, wells were washed with distilled water and left to dry. Subsequently, 300 µL of 95% ethanol was added to each well for 15 min with the lid closed. Gentle pipetting was performed to mix the content properly, and 150 µL of ethanol/crystal violet solution was transferred into a new 96-well plate and the optical density was measured at 630 nm using a Multiskan Sky Microplate Spectrophotometer. The experiments were performed in triplicates, and one well served as a medium control (blank).

The percent biofilm inhibitory activity was calculated using the formula: [(*C − B*) *−* (*T − B*)/(*C − B*)]∗100%, where *B* = absorbance of blank (only TSB), *C* = absorbance of the control (biofilm, no treatment), and *T* = absorbance of the test (biofilm and treatment) [36]. In this experiment, absolute ethanol (96%) was used as a positive control, which exhibited 100% inhibition of bacterial growth/biofilm formation.

### 4.7. RNA Extraction, cDNA Synthesis, and qPCR Analysis of the Genes Involved in Biofilm Formation

In the next step, we employed a quantitative-PCR (qPCR) assay to analyze the effect of natural compounds on the expression of genes involved in biofilm formation [37]. In the first step, bacterial RNA extraction was performed. For this, *S. aureus* cultures were grown in TSB with either compounds or DMSO in clean autoclaved 1.5 mL micro-centrifuge tubes. Bacterial cultures were harvested after overnight incubation at 37 °C. The bacterial cells were rinsed with sterile PBS and centrifuged at 4000 rpm for 10 min to get a clear cell pellet. Approximately 5 × 10^6^ cells were suspended in 500 µL of RLT buffer containing 0.45–0.55 mm glass beads and vortexed at maximum speed for 45 s. The RNA was extracted from bacterial cell lysate using the RNeasy mini kit (QIAGEN, Hilden, Germany). The RNA concentration and purity were quantified by nanodrop, and RNA was stored at −80 °C until further use. RNA was reverse-transcribed by using an M-MLV reverse transcriptase kit (Promega, Madison, WI, USA). The RNA template (5 µL) was mixed with 1 µL OligodT (0.5 µg/µL), 1 µL dNTPs 10 µM, and 8 µL nuclease-free water, and incubated on a pre-heated block at 65 °C for 5 min. At the end of incubation, the reaction mixture was immediately chilled on ice for 5 min, then briefly centrifuged to bring the contents down to the bottom of the tube. This reaction was combined with a reaction mixture containing 4 µL M-MLV RT 5 × reaction buffer and 1 µL M-MLV reverse transcriptase (10,000 U) to get the volume up to 20 µL. This reaction mixture was incubated at 50 °C for 30 min, 85 °C for 5 min, and 4 °C and held there in an Eppendorf (Hamburg, Germany) thermal cycler.

The cDNA samples were used in a qPCR assay, where gene-specific primers (Table 3) were used to measure the changes in expression levels of *ica-A, fnb-B, clf-A,* and *cna* genes (involved in *S. aureus* biofilm formation) [14] in untreated and treated samples. The 16s-rRNA served as a housekeeping gene and was also used to normalize the results.

For qPCR analysis, a 20 µL reaction mixture was prepared using the following recipe: 2 µL of cDNA, 0.6 µL (10 pmol/µL) of forward and reverse primers, and 10 µL of Evergreen qPCR master mix (ABM, Richmond, BC, Canada). The qPCR reaction was run on a CFX96™ Real-Time PCR System (BIO-RAD, Hercules, CA, USA) using a thermo-cycling protocol: 5 min at 95 °C, 40 cycles of 20 s at 95 °C, 20 s at 60 °C, and 20 s at 72 °C. A melt curve (55 °C–95 °C) analysis was performed at the end of the 40 cycles to verify the identity of the PCR products. All reactions were run in triplicate. The Delta CT value was calculated by subtracting the average Ct for the housekeeping gene from the average Ct for the gene of interest [38]. To determine the significant difference in mean gene expression, an independent *t*-test was performed in GraphPad prism, where *p* < 0.05 was taken as a statistically significant value.

## 5. Conclusions

This study evaluated the significant downregulation of gene expression by phenyl propenes and phenolic aldehyde that can deactivate bacterial adhesion and reduce biofilm production in *S. aureus.* Therefore, we can conclude that these bioactive molecules can suppress the virulence factors and may serve as drug candidates for antimicrobial and anti-biofilm agents.

## Figures and Tables

**Table 1:** **Antibiogram of *S. aureus* clinical isolates.** Eight regularly prescribed antibiotics were tested against *S. aureus* isolates for antibiotic resistance/susceptibility.

Antibiotics	Sensitive % (N)	Intermediate % (N)	Resistant % (N)
Ampicillin 10 µg (AMP)	34.3 (11)	31.2 (10)	34.3 (11)
Gentamicin 120 µg (CN)	93.7 (30)	0	6.2 (2)
Chloramphenicol 30 µg (C)	90.6 (29)	3.1 (1)	6.2 (2)
Ciprofloxacin 5 µg (CIP)	34.3 (11)	12.5 (4)	53.1 (17)
Amikacin 30 µg (AK)	81.2 (26)	12.5 (4)	6.2 (2)
Clindamycin 2 µg (DA)	65.6 (21)	21.8 (7)	12.5 (4)
Streptomycin 10 µg (S)	84.3 (27)	0	15.6 (5)
Cephalothin 30 µg (KF)	81.2 (26)	0	18.5 (6)

**Table 2:** Minimum inhibitory concentration (MIC) and percentage biofilm inhibition exhibited by the natural compounds.

Isolates	S11		S12		S31		S24		S25	
Natural Compounds	Biofilm Inhibition (%)	MIC (mg/mL)	Biofilm Inhibition (%)	MIC (mg/mL)	Biofilm Inhibition (%)	MIC (mg/mL)	Biofilm Inhibition (%)	MIC (mg/mL)	Biofilm Inhibition (%)	MIC (mg/mL)
**SALI**	70.66	1	75.07	1	92.52	1	72.67	30	88.59	12.5
**VAN**	72.99	1	73.37	1	87.38	55	70.15	40	83.77	55
**A-MT**	70.15	27.5	73.44	30	84.58	25	70.3	100	85.53	30
**A-BT**	72.34	1	58.31	1	85.98	0.75	72.03	1	89.91	5
**NNDC**	68.91	2.5	66.25	5	69.16	21	67.84	150	58.33	100
**4A3M**	72.48	1	68.92	1	85.05	12.5	55.8	1	89.47	20
**A-MCA**	66.57	1	67.43	1	15.88	5	53.17	2.5	87.38	5
**3H4M**	68.91	2.5	72.55	2.5	91.59	17.5	63.09	7.5	81.58	160
**4H35**	67.52	5	72.77	2.5	92.99	15	69.07	25	90.35	25
**T4N**	72.99	1	72.03	1	88.31	1	71.16	250	87.28	250
**EUG**	70.51	7.5	72.63	7.5	38.32	100	58.26	100	16.22	100
**Ethanol** **(+ve control)**	100	-	100	-	100	-	100	-	100	-
**Blank media (−ve control)**	0	-	0	-	0	-	0	-	0	-

Key: SALI: salicylaldehyde, VAN: Vanillin, A-MT: α-methyl- trans-cinnamaldehyde, A-BT: α-bromo-trans-cinnamaldehyde, NNDC: N, N-dimethyl-cinnamaldehyde, 4A3M: 4-acetoxy-3-methoxycinnamaldehyde, A-MCA: α-methyl-cinnamic acid, 3H4M: 3-hydroxy-4-methoxycinnamic acid, 4H35: 4-hydroxy-3,5-dimethoxycinnamic acid, T4N: trans-4-nitrocinnamic acid, EUG: Eugenol.

**Table 3:** Name of target genes and respective primer sets used to quantify mRNA levels in qPCR.

Gene	Forward Primer (5′ to 3′)	Reverse Primer (5′ to 3′)
*16S rRNA*	GGGACCCGCACAAGCGGTGG	GGGTTGCGCTCGTTGCGGGA
*icaA* (intercellular adhesion gene)	GAGGTAAAGCCAACGCACTC	CCTGTAACCGCACCAAGTTT
*fnbA* (fibronectin-binding protein A)	AAATTGGGAGCAGCATCAGT	GCAGCTGAATTCCCATTTTC
*clfA* (clumping factor A)	ACCCAGGTTCAGATTCTGGCAGCG	TCGCTGAGTCGGAATCGCTTGCT
*cna* (collagen binding protein)	AATAGAGGCGCCACGACCGT	GTGCCTTCCCAAACCTTTTGAGC

## Data Availability

Not applicable.

## Supplementary Materials

The following supporting information can be downloaded at: https://www.mdpi.com/article/10.3390/molecules27206874/s1. Supplementary Figure S1: Air-liquid interface coverslip assay for biofilm analysis. The images show dense staining and microbial aggregation, consistent with biofilm formation, on coverslips by all isolates tested (S1–S32). Supplementary Table S1: ImageJ analysis for stain color intensity (biofilm formation) showing mean scores with SD for each isolate.
