# Supplementary material for: Analysis of the Antimicrobial and Anti-Biofilm Activity of Natural Compounds and Their Analogues against Staphylococcus aureus Isolates
Source: Molecules. 2022 Oct 13;27(20):6874. doi: 10.3390/molecules27206874 (PMC9610881; doi:10.3390/molecules27206874)

Supplementary Table S1

Image of clinical isolate #	Biofilm stain color intensity (mean scores with SD)
S1	80.33 ±28.66
S2	71.67 ± 36.33
S3	101 ± 35
S4	100 ± 33.33
S5	57.67 ± 28.66
S6	67.33 ± 30.66
S7	102.66 ± 27.33
S8	123 ± 45
S9	94 ± 57
S10	104.33 ± 55.33
S11	75 ± 46
S12	88.33 ± 41
S13	100.66 ± 50.67
S14	77.33 ± 22
S15	140.66 ± 42.33
S16	73.33 ± 23.33
S17	71 ± 26.67
S18	92.67 ± 53.67
S19	69 ± 20.33
S20	112.33 ± 48
S21	88.67 ± 43
S22	61.33 ± 27
S23	114 ± 51
S24	46.67 ± 30.33
S25	67.66 ± 30
S26	67 ± 21.67
S27	79.67 ± 28.67
S28	91.66 ± 48
S29	89 ± 46
S30	123.67 ± 68.33
S31	142.33 ± 55.67
S32	66 ± 34

Supplementary Figure S1

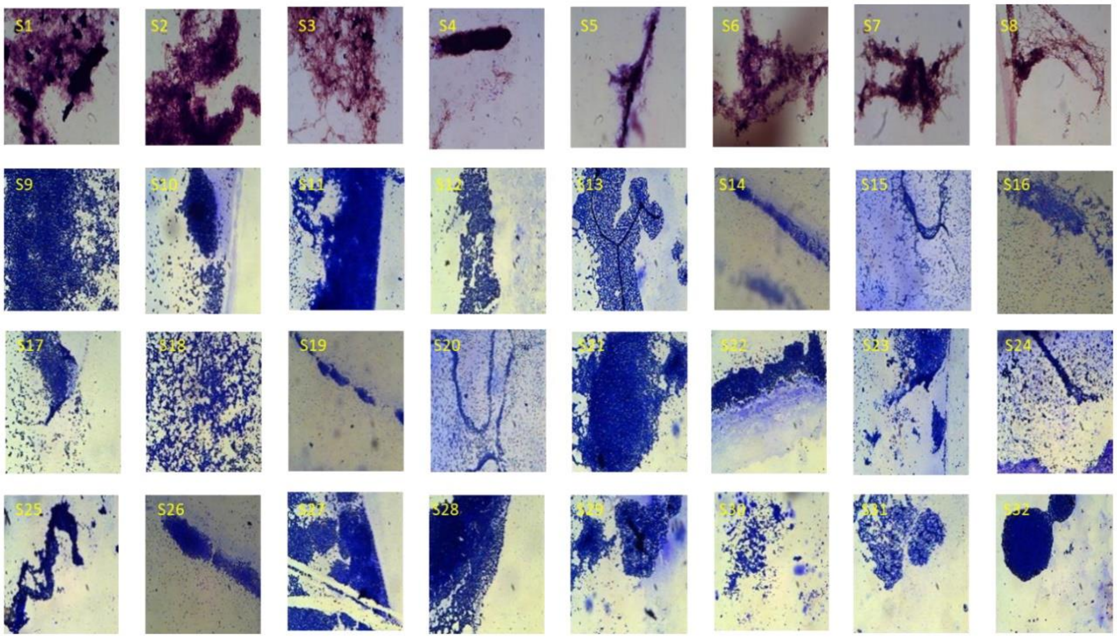

Supplement: Supplementary file 1 [file molecules-27-06874-s001.zip › molecules-1918667-supplementary.pdf]
